# Poor association between tendon structure and self-reported symptoms following conservative management in active soldiers with mid-portion Achilles tendinopathy

**DOI:** 10.1136/military-2022-002241

**Published:** 2022-10-21

**Authors:** M A Paantjens, P H Helmhout, F J G Backx, M T A W Martens, J P A van Dongen, E W P Bakker

**Affiliations:** 1 Sports Medicine Centre, Training Medicine and Training Physiology, Royal Netherlands Army, Utrecht, The Netherlands; 2 Department of Rehabilitation, Physical Therapy Science and Sports, University Medical Centre Utrecht, Utrecht, The Netherlands; 3 Centre of Excellence, Training Medicine and Training Physiology, Royal Netherlands Army, Utrecht, The Netherlands; 4 Fontys University of Applied Science, School of Allied Health Professions, Eindhoven, The Netherlands; 5 National Institute of Musculoskeletal Ultrasound, Vianen, The Netherlands; 6 Department Epidemiology and Data Science, Division EPM, Amsterdam UMC Locatie AMC, Amsterdam, The Netherlands

**Keywords:** sports medicine, ultrasonography, foot & ankle

## Abstract

**Introduction:**

Mid-portion Achilles tendinopathy (mid-AT) is currently the preferred term for persistent Achilles tendon pain, defined as located 2–7 cm proximal to the calcaneus, and with loss of function related to mechanical loading. Histologically, mid-AT is considered to represent a degenerative condition. Therefore, monitoring of tendon structure additional to pain and function may be warranted, to prevent progression of degeneration or even tendon rupture. The aim of this study was to determine the association between pain and function, relative to the Achilles tendon structure, in soldiers treated with a conservative programme for mid-AT.

**Methods:**

A total of 40 soldiers (40 unilateral symptomatic tendons) were included in this study. Pain and function were evaluated with the Victorian Institute of Sports Assessment -Achilles (VISA-A) questionnaire. Tendon structure was quantified using ultrasound tissue characterisation (UTC). We quantified both the Achilles tendon mid-portion (2–7 cm) and the area of maximum degeneration (AoMD) within the tendon mid-portion. VISA-A and UTC measurements were taken at baseline and after 26 weeks of follow-up. Spearman’s rho was used to determine the correlation between VISA-A and UTC. Correlations were calculated for baseline, follow-up and change score values.

**Results:**

Negligible correlations were found for all analyses, ranging from −0.173 to 0.166 between mid-portion tendon structure and VISA-A, and from −0.137 to 0.150 between AoMD and VISA-A. While VISA-A scores improved, on average, from 59.4 points at baseline to 93.5 points at follow-up, no detectable improvement in aligned fibrillar structure was observed in our population.

**Conclusion:**

Pain and function are poorly associated with Achilles tendon structure in soldiers treated with a conservative programme for mid-AT. Therefore, we advise clinicians to use great caution in communicating relationships between both clinical entities.

**Trial registration number:**

NL69527.028.19.

**WHAT IS ALREADY KNOWN ON THIS TOPIC**

- Achilles tendon structure appears poorly associated with disease severity and prognosis in non-military populations treated with eccentric loading exercises for mid-portion Achilles tendinopathy (mid-AT).

**WHAT THIS STUDY ADDS**

- Both the Achilles tendon mid-portion (2–7 cm) and the area of maximum degeneration (AoMD) within the tendon mid-portion are poorly associated with pain and function in soldiers treated with extra corporeal shockwave therapy (ESWT) and load management for mid-AT.
- In our population, a combined treatment of ESWT and exercise did neither improve aligned fibrillar structure in the Achilles tendon mid-portion nor in the AoMD within a 26-week period.

**HOW THIS STUDY MIGHT AFFECT RESEARCH, PRACTICE OR POLICY**

- In soldiers with mid-AT, great caution is advised in communicating (causal) relationships between tendon structure on the one hand and pain and function on the other hand.

## Introduction

Achilles tendinopathy (AT) is currently the preferred term for persistent Achilles tendon pain and loss of function related to mechanical loading.^1^ The treatment is initially conservative.^2^ AT has been reported to occur in a wide age range of 20–69 years, with peaks between 40 and 59 years.^3^ Active individuals are most frequently affected, particularly runners, with life-time prevalences ranging up to 52%.^2^ AT is also common in soldiers, significantly impacting activity levels and military operational readiness.^4^

AT can be divided into mid-portion Achilles tendinopathy (mid-AT) and insertional Achilles tendinopathy.^2^ Mid-AT, defined as located 2–7 cm proximal to the calcaneus, is an isolated tendinopathy, generally considered to represent a degenerative condition.^3 5 6^ In tendinopathy, tendon loading may cause progressive degeneration,^6^ in rare occasions (4%) ultimately leading to a tendon rupture.^3^ However, tendon loading can also be an anabolic stimulus to improve tendon structure.^6^

Clinicians should primarily assess the domains of pain and function when evaluating patients with mid-AT, using the Victorian Institute of Sports Assessment-Achilles (VISA-A) questionnaire as the preferred patient reported outcome measure.^2 7^ Additional monitoring of a tendon’s structural response to biomechanical loading may also be of importance, especially in physically demanding professions, such as soldiers, for whom the rare event of an Achilles tendon rupture can have serious consequences.

Ultrasound tissue characterisation (UTC) is an imaging technique to visualise and quantify the mid-portion Achilles tendon structure.^8^ UTC discriminates four echo-types (I–IV) within the Achilles tendon matrix. Combined echo-types I+II represent aligned fibrillar structure, whereas echo-types III+IV can be seen as disorganised tendon structure. UTC can be used to monitor load^9 10^ or to evaluate treatment.^11 12^

While growing evidence indicates that tendon structure should not be used to explain the presence or severity of current and future symptoms in AT, the evidence is still conflicting.^8 10–13^ With regard to the clinical applicability of UTC, we aimed to determine the association between pain and function, relative to the mid-portion Achilles tendon structure, in soldiers treated with a conservative treatment programme for mid-AT.

## Methods

### Study setting

The study was conducted at the Sports Medicine Centre of the Department of Training Medicine and Training Physiology of the Royal Netherlands Army, Utrecht, the Netherlands. This centre is a secondary care facility for soldiers that predominantly focuses on researching and treating persistent musculoskeletal health problems.

### Eligibility criteria

Consecutive patients, referred to the Sports Medicine Centre for AT between July 2019 and January 2021, were potentially eligible for inclusion based on the following criteria: (1) military personnel in active duty (18–60 years); (2) a clinically established diagnosis of mid-AT^2^ and (3) symptoms for 2 months or more. In case of bilateral symptoms, only the side with the lowest VISA-A score was included into the analysis.

Participants were excluded if they reported concomitant insertional Achilles tendinopathy; or on the presence of factors that may have adversely affected tendon structure: (1) signs of a complete Achilles tendon rupture; (2) prior surgery to the Achilles tendon; (3) use of statins, fluoroquinolones or corticosteroids^14^ and (4) a previous diagnosis of rheumatoid arthritis, diabetes mellitus or psoriasis.^14^ All participants were recruited by the main researcher (MP, physical therapist). Prior to inclusion, each participant provided written informed consent for anonymous use of their data.

### Patient evaluation

#### Patient characteristics

At baseline, the following patient characteristics were retrieved: age (years), height (cm), weight (kg), body mass index (BMI, %), gender (male/female) and symptom duration (months).

#### Baseline and follow-up measurements

**Figure 1:**
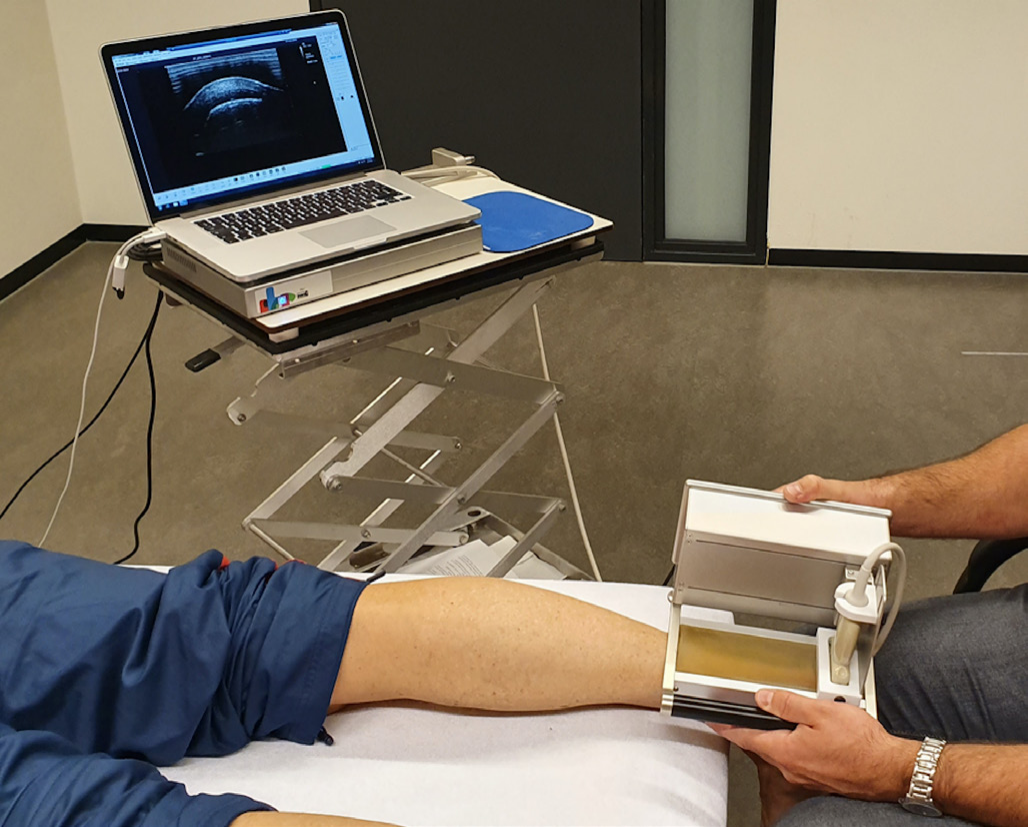
Ultrasound tissue characterisation scanning of the Achilles tendon with the patient in a prone position.

Measurements were performed at baseline and during follow-up at week 26, and consisted of a written VISA-A questionnaire^7^ and a UTC scan (Figure 1).^8^

VISA-A is a validated, disease-specific instrument to assess pain, function in daily living and sporting activity.^7^ The sum score on the VISA-A can range from 0 to 100 points, where 100 represents a perfect asymptomatic score. All participants independently completed the VISA-A questionnaire prior to the UTC investigation, in order to avoid the imaging outcome to influence the VISA-A scoring.

UTC scans were collected and processed according to a standardised protocol, which has shown excellent intra-rater and inter-rater reliability in the same patient group used for this study.^15^ A single experienced examiner in UTC (MP) collected the UTC scans. Images were acquired with a 12 MHz linear ultrasound transducer (Terason 12L5 Smartprobe, Vermon, France), using Terason software (t2000+OEM). This transducer was embedded in a motorised tracking device (UTC tracker, UTC imaging, 6171GD Stein, The Netherlands, serial no. UTC-201-041). An independent researcher (MTAWM, physical therapist), blinded to the VISA-A scoring, processed the UTC scans, aiming to quantify: (1) the mid-portion Achilles tendon structure (2–7 cm) and subsequently (2) the area of maximum degeneration (AoMD) within the tendon mid-portion.^15^

### Rehabilitation programme

**Figure 2:**
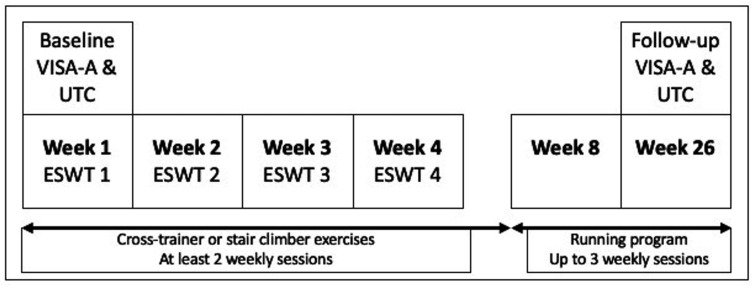
The rehabilitation programme of soldiers with mid-portion Achilles tendinopathy. In the first 4 weeks, all soldiers received weekly sessions of extracorporeal shockwave therapy. During the first 8 weeks, all soldiers performed an individualised exercise programme on a stair climber or cross-trainer, followed by a return to running programme from week 8 to 26.

The rehabilitation programme of 26 weeks (figure 2) consisted of patient education,^2^ extracorporeal shockwave therapy (ESWT), exercise on a cross-trainer or stair climber and a return to running programme (online supplemental file 1).^16^

10.1136/military-2022-002241.supp1 Supplementary data

### Sample size calculation

Based on prior research,^8 10 11^ for sample size calculation we assumed a low correlation of .45 between UTC and VISA-A scores.^17^ With a default alpha of .05 and a statistical power of .80, a sample size of 36 participants was calculated. Taking into account a 10% loss to follow-up, we included a total of 40 active soldiers for this study.

### Statistics

Baseline characteristics of our study population were presented with appropriate measures of central tendency and dispersion. For tendon structure, aligned fibrillar structure (echo-types I+II) and disorganised tendon structure (echo-types III+IV) were used as the two outcomes.^8^ Both echo-type combinations were expressed as a percentage of the total Achilles volume analysed with UTC.

Pearson’s correlation coefficients were chosen to determine the strength and direction of the association between UTC and VISA-A scores.^17^ When data were not normally distributed, Spearman’s rank correlation coefficients were used.^17^ Correlation coefficients were interpreted either as: negligible (0.00 to 0.30), low (0.30 to 0.50), moderate (0.50 to 0.70), high (0.70 to 0.90) and very high (0.90 to 1.00).^17^

Correlations between VISA-A scores and UTC were calculated at baseline and follow-up, as well as for pre-post change scores, in order to evaluate the responsiveness of UTC.

All analyses were performed using SPSS (IBM SPSS Statistics for Windows, V.25.0, IBM, Armonk, New York, USA).

### Ethical considerations

The data were collected as part of an observational study (https://www.toetsingonline.nl/to/ccmo_search.nsf/Searchform?OpenForm, file number ToetsingOnline NL69527.028.19), aiming to evaluate ESWT and load management in service members suffering from mid-AT.

## Results

A total of 40 patients were included in this study, of which 12 reported bilateral symptoms. No participants were lost to follow-up. Patient characteristics are presented in Table 1.

**Table 1:** Patient characteristics of active soldiers with mid-AT

Characteristics	Total group (n=40) Mean±SD
Age (years)	40.1±9.4
Height (cm)	185.1±5.9
Weight (kg)	93.8±13.2
Body mass index (%)	27.4±3.3
Gender (male/female)	38/2
Duration of symptoms (months)	13.0±16.5

n, number; SD, standard deviation.

The mean VISA-A score improved from baseline (59.4±SD 17.3, range 15–86) to follow-up at 26 weeks (93.5±SD 9.8, range 48–100).

The mean UTC echo-types for the Achilles tendon mid-portion and the AoMD are displayed in Table 2. Echo-type change scores were calculated from baseline to follow-up. We determined their clinical relevance by comparing the values with the minimal detectable changes (MDC) calculated for this particular cohort.^15^

**Table 2:** Mean UTC echo-types of the participants Achilles tendon mid-portion and the AoMD at baseline, and during follow-up at 26 weeks

Echo-type	Mid-portion Total group (n=40) Mean±SD (min-max)		AoMD Total group (n=40) Mean±SD (min-max)	
	Baseline	Follow-up	Baseline	Follow-up
I (%)	48.3±11.9 (27.3–75.2)	51.4±11.3 (28.8–75.6)	37.1±11.9 (14.7–64.8)	40.7±11.8 (17.0–65.1)
II (%)	19.4±4.6 (10.5–32.9)	21,0±4.5 (10.5–32.1)	22.2±7.4 (12.2–42.9)	22.6±7.1 (9.8–39.9)
III (%)	19.1±8.7 (2.6–35.5)	16.7±8.7 (1.9–37.8)	26.6±11.9 (1.5–49.7)	23.7±11.5 (2.8–49.5)
IV (%)	13.1±6.6 (1.9–29.2)	10.8±5.3 (1.4–25.6)	13.8±6.7 (0.4–24.3)	12.4±5.9 (0.8–27.3)
Total	100%	100%	100%	100%
I+II (%)	67.8±14.4 (40.8–95.3)	72.4±13.2 (42.5–96.6)	59.2±17.0 (30.0–98.1)	63.3±16.4 (27.4–96.5)
III+IV (%)	32.2±14.4 (4.7–59.3)	27.6±13.2 (3.3–57.5)	40.4±17.5 (1.9–70.0)	36.2±16.4 (3.6–72.5)
Total	100%	100%	100%	100%

Echo-types I, II, III and IV are expressed as a percentage of the analysed Achilles tendon volume. Combined, the echo-types I+II represent aligned fibrillar structure, and the echo-types III+IV disorganised tendon structure.

AoMD, area of maximum degeneration (one slide in the Achilles tendon mid-portion with the lowest representation of echo-type I); max, maximum; min, minimum; n, number; SD, standard deviation; UTC, ultrasound tissue characterisation.

In the mid-portion, aligned fibrillar structure (echo-type I+II) showed a 4.6% improvement, which was below the MDC of 5.9%. Disorganised tendon structure (echo-type III+IV) in the mid-portion decreased with 4.6%, exceeding the MDC of 4.2%.

In the AoMD, aligned fibrillar structure improved with 4.1%, not exceeding the MDC of 9.8%. Disorganised tendon structure in the AoMD decreased with 4.2%, also not exceeding the MDC of 10.0%.

Regarding the changes of individual mid-portion echo-types, echo-type IV decreased with 2.3%. This was the only individual echo-type that showed a change score above the MDC (1.9%). All other change scores were below this threshold: echo-type I 3.1% (MDC 4.7%), echo-type II 1.6% (MDC 2.5%) and echo-type III 2.4% (MDC 3.6%).

No AoMD change scores of individual echo-types exceeded the MDC: echo-type I 3.6% (MDC 4.8%), echo-type II 0.4% (MDC 8.2%), echo-type III 2.9% (MDC 6.8%) and echo-type IV 1.4% (MDC 4.6%).

Baseline, follow-up and change score correlations between VISA-A and UTC are reported in Table 3.

**Table 3:** Correlations between the VISA-A scores and UTC-typing at baseline, during follow-up after 26 weeks and with regard to the change scores

	VISA-A baseline	VISA-A follow-up	VISA-A difference
Mid-portion baseline ET1+2	ρ=0.166, sig. (two-tailed) 0.306		
Mid-portion follow-up ET1+2		ρ=0.046, sig. (two-tailed) 0.778	
Mid-portion difference ET1+2			ρ=−0.013, sig. (two-tailed) 0.935
Mid-portion baseline ET3+4	ρ=−0.173, sig. (two-tailed) 0.287		
Mid-portion follow-up ET3+4		ρ=−0.048, sig. (two-tailed) 0.769	
Mid-portion difference ET3+4			ρ=0.018, sig. (two-tailed) 0.912
AoMD baseline ET1+2	ρ=0.131, sig. (two-tailed) 0.422		
AoMD follow-up ET1+2		ρ=0.150, sig. (two-tailed) 0.354	
AoMD difference ET1+2			ρ=0.024, sig. (two-tailed) 0.882
AoMD baseline ET3+4	ρ=−0.126 sig. (two-tailed) 0.422		
AoMD follow-up ET3+4		ρ=−0.137, sig. (two-tailed) 0.398	
AoMD difference ET3+4			ρ=−0.024, sig. (two-tailed) 0.885

AoMD, area of maximum degeneration (one slide in the Achilles tendon mid-portion with the lowest representation of ET I); ET, echo-type; sig., significance; UTC, ultrasound tissue characterisation; VISA-A, Victorian Institute of Sports Assessment-Achilles questionnaire; ρ, Spearman’s rho.

## Discussion

### Association between the VISA-A and tendon structure

The primary objective of this study was to investigate the association between pain and function, relative to tendon structure, in soldiers treated with a conservative programme for mid-AT. In tendinopathy, improvements in pain and function generally precede the much slower restoration of tendon structure.^18^ Therefore, we hypothesised that the AoMD would show a stronger association with VISA-A than the tendon mid-portion, since relative changes in tendon structure can be expected to be larger than in the mid-portion analysis. This turned out not to be the case as we found negligible correlations for all analyses, indicating that tendon structure is poorly associated with pain and function in soldiers with mid-AT.

Our findings are supported by two non-military studies evaluating the association between pain and function versus tendon structure, in mid-AT patients.^11 12^ In the first study, de Vos *et al* 12 evaluated subjects who followed an eccentric loading programme, reporting negligible correlations between changes scores on VISA-A and echo-types I+II from baseline to follow-up at 24 weeks.^12^ Baseline echo-types I+II also showed negligible correlations with VISA-A scores after 24 weeks.^12^ In the second study, de Jonge *et al* 11 evaluated eccentric loading combined with either a platelet-rich plasma injection or a saline injection,^11^ also reporting no associations between VISA and UTC.^11^ Both studies concluded that tendon structure was not related to disease severity or prognosis in mid-AT.^11 12^

In tendinopathy, the exact pathophysiology and the source of nociception are currently unknown.^2 6 19^ The fact that recovery of tendon structure and improvement in pain and function do not follow the same pace may partly explain the poor associations.^18^ Positive changes in the pain system following tendinopathy treatment can already occur in 2 weeks, with results peaking at 12 weeks,^20^ while full restoration of tendon structure takes considerably longer, from 24 weeks^11^ up to several years.^18^

### Mean improvement on the VISA-A

The VISA-A score improved, on average, from 59.4 points at baseline to 93.5 points at follow-up. Although scores can range from 0 to 100 points, a score of 90 points is reported to represent full recovery from mid-AT.^21^ A recent meta-analysis concluded that VISA-A scores may be expected to improve by approximately 21 points, following exercise interventions for mid-AT.^20^ We have found a considerably higher mean VISA-A improvement of 34.1 points.

Several factors may explain the large improvements found in our study. One possible explanation is that we used a combination of ESWT and exercise in our rehabilitation programme, as this combination is suggested to achieve higher VISA-A scores than exercise alone.^22^ It is also possible that an above average treatment compliance of our study group, consisting of generally sports-minded, instruction-compliant soldiers, may have positively influenced the results. Finally, we cannot rule out the potential influence of additional ultrasonography in our programme. When subjects were in doubt whether continued or progressive exercise adversely affected their tendon structure, a grey scale ultrasound was performed to rule out any tendon abnormalities. Grey scale ultrasound did not reveal any adverse tendon changes in the vast majority of the cases. It is possible that visual confirmation of unchanged or uncompromised tendon structure, along with patient education, may have positively influenced illness perceptions, contributing to higher VISA-A scores. Illness perceptions are reported to have a cross-sectional relationship with musculoskeletal pain.^23^

### Mean changes of Achilles tendon structure

We have only observed detectable changes in the mid-portion analysis, with both disorganised tendon structure and echo-type IV barely exceeding the MDC by 0.4%. Despite an above-average increase in the mean VISA-A score, no improvement in aligned fibrillar tendon structure was observed after 26 weeks.

Our findings contradict the results of an in vivo study^24^ and an in vitro study^25^ that suggest ESWT-induced tendon structure improvements, but are in line with a study by de Vos *et al*,^12^ who reported no increase of aligned fibrillar structure following an eccentric loading programme after 24 weeks. Contrastingly, de Jonge *et al* 11 reported a mean improvement in aligned fibrillar structure of 11% following an eccentric loading programme. It should be acknowledged that in the latter study, patients additionally received either a platelet-rich plasma injection or a saline injection.

### Rehabilitation programme

A total of 9 out of 40 participants included in this study had not undergone previous treatment. The other 31 patients were referred for ESWT due to unsatisfactory results in primary care, where they received various interventions, that is, non-steroidal anti-inflammatory drugs, ankle mobilisation, calf stretching exercises, massage and gait retraining. Tendon loading exercises had been a part of the previous treatment in 28 participants. Although these exercises currently represent the standard of care for mid-AT,^2^ a large number of patients does not seem to respond adequately, and up to half of all patients seeking alternative treatment.^26^

### Clinical applicability

As tendon structure appears poorly associated with pain and function in mid-AT, we recommend assessing both clinical entities separately. We strongly advise clinicians to use great caution in communicating (causal) relationships between tendon structure and pain and function in soldiers suffering from mid-AT, as our results cannot support this in any way. It is our belief that in physically highly active populations, like soldiers, assessment of Achilles tendon structure should be used to evaluate load, or to evaluate interventions targeting tendon structure, and also to prevent potential structural damage to Achilles tendons.^3^

### Limitations

A number of potential limitations may have influenced the results of this study.

First, both the UTC scans and VISA-A scores were collected by the same researcher. It is unlikely that this has influenced the outcomes, as the VISA-A is a self-completing questionnaire,^21^ and the UTC scanning procedure is highly standardised.^15^

Second, for reasons of standardisation, and due to positive clinical experiences over the years, we have chosen to replace traditional tendon loading exercises^2^ by a stair climber or cross-trainer in our study. This appears to have had no major negative effects on pain and function, as the mean VISA-A score at follow-up (93.5 points) indicates complete recovery from mid-AT.^21^ We did not observe detectable improvements in aligned fibrillar structure after 26 weeks. Whether this would have been the case if we had incorporated traditional tendon loading exercises into our rehabilitation programme is questionable, as there is currently limited and conflicting evidence regarding this topic.^11 12^ Possibly, our follow-up of 26 weeks was too short to observe improvement of aligned fibrillar structure.^18^

Third, while tendon loading exercises had been part of a previous treatment programme in 28 participants included in our study, it should be acknowledged that 13 of those 28 participants had not completed a full 12-week programme as recommended by the clinical guideline.^2^ Because improvements in pain and function are reported to peak at 12 weeks following inception of such a programme,^20^ it is quite possible that for some of these 13 participants tendon loading would have been more effective if they had completed the full 12 weeks.

Possible future studies could compare traditional tendon loading exercises with exercise on a stair climber or cross-trainer in mid-AT, or explore the relationship between the use of ultrasound, illness perceptions and patient-reported outcomes evaluating pain and function in mid-AT.

## Data Availability

Data are available on reasonable request. The data supporting the findings of this study are available from the corresponding author on reasonable request.
