# Supplementary material for: Poor association between tendon structure and self-reported symptoms following conservative management in active soldiers with mid-portion Achilles tendinopathy
Source: BMJ Mil Health. 2022 Oct 21;170(4):331–6. doi: 10.1136/military-2022-002241 (PMC11287647; doi:10.1136/military-2022-002241)
Supplement: Supplementary data [file military-2022-002241supp001.pdf]

Supplementary File 1. The rehabilitation program of soldiers with mid-portion Achilles tendinopathy.

Patient education
First patient education was provided, emphasizing the importance of an active treatment strategy, the nature of the pathological condition, pain education, psychosocial factors, load-management, the (long-term) prognosis, and return to full (sports) activities.

Focused extracorporeal shockwave therapy (F-ESWT)
All participants received weekly sessions of ESWT in the first 4 weeks. ESWT was administrated by MP, while subjects lay prone, with their feet hanging freely over the examining table. This allowed the ipsilateral foot to be fixed in maximum dorsiflexion, to make the tendon easily accessible for the shockwave applicator. Each ESWT session consisted of 2000 shocks, distributed evenly over the painful mid-portion area, with a frequency of 8 shocks per second. Immediately after initiating the ESWT treatment, the therapist rapidly increased the intensity to the level the subject considered tolerable for the treatment duration of approximately 4 minutes (up to a total energy density of 0.82mJ/mm2).

Exercise interventions
Following a one on one instruction, the exercise interventions were carried out individually. Exercise related pain was allowed up to a threshold of 5 points on a numeric pain rating scale (range 0-10). Because running is known to be provocative of mid-AT symptoms in the majority of cases in our population, it was temporarily replaced with an individualized and gradual build-up program on a stair climber or cross-trainer from week 1 to 8 of the rehabilitation program (Figure 2). In our experience this is generally able to be conducted within the advised pain threshold. Exercise on a stair climber or cross-trainer was performed at least 2 times a week, up to one hour per session, during which the participants were instructed to actively lift their heels in order to facilitate plantar flexor activity.
From week 8 to 26, the running program from the Dutch Heart Foundation was performed, up to a maximum of 3 weekly sessions.
